# Correction: Facile synthesis of amino-modified magnetic covalent organic framework for the efficient extraction and determination of anionic azo dyes in carbonated beverages

**DOI:** 10.1007/s44211-024-00584-w

**Published:** 2024-05-07

**Authors:** Shuping Guan, Hao Wu, Wanming Lin, Yaxin Chen, Zhuliang Wang

**Affiliations:** 1 College of New Energy and Materials Engineering, Shanxi Electronic Science and Technology University, Linfen, China; 2 School of Chemistry and Materials Science of Shanxi Normal University, Key Laboratory of Magnetic Molecules and Magnetic Information Materials of Ministry of Education, Shanxi Normal University, Taiyuan, China; 3 Shanxi Yitiantai Testing Technology Co., Ltd, Linfen, China; 4 College of Intelligent Manufacturing, Shanxi Electronic Science and Technology University, Linfen, China

**Correction: Analytical Sciences** 10.1007/s44211-024-00561-3

The article, “Facile synthesis of amino‑modified magnetic covalent organic framework for the efficient extraction and determination of anionic azo dyes in carbonated beverages”, written by Shuping Guan et al., was originally published online on the publisher’s internet portal on 04 April 2024 with Open Access under a Creative Commons Attribution (CC BY) license 4.0.

With the authors' decision to cancel Open Access the copyright of the article changed on 19 April 2024 to ©The Author(s), under exclusive licence to The Japan Society for Analytical Chemistry 2024 with all rights reserved.

